# Development and validation of a novel prognosis prediction model for patients with myelodysplastic syndrome

**DOI:** 10.3389/fonc.2022.1014504

**Published:** 2022-10-12

**Authors:** Haiping Liang, Yue Feng, Yuancheng Guo, Jinli Jian, Long Zhao, Xingchun Luo, Lili Tao, Bei Liu

**Affiliations:** ^1^ The First Clinical Medical College of Lanzhou University, Lanzhou, China; ^2^ Department of Blood Transfusion, the Second Affiliated Hospital of Xi’an Jiaotong University, Xi’an, China; ^3^ Department of Hematology, The First Hospital of Lanzhou University, Lanzhou, China; ^4^ Department of Hematology, Xi’an Central Hospital, Xi’an, China

**Keywords:** Myelodysplastic Syndrome, somatic mutation, prognosis, nomogram, risk stratification

## Abstract

**Background:**

Somatic mutations are widespread in patients with Myelodysplastic Syndrome (MDS) and are associated with prognosis. However, a practical prognostic model for MDS that incorporates somatic mutations urgently needs to be developed.

**Methods:**

A cohort of 201 MDS patients from the Gene Expression Omnibus (GEO) database was used to develop the model, and a single-center cohort of 115 MDS cohorts from Northwest China was used for external validation. Kaplan-Meier analysis was performed to compare the effects of karyotype classifications and gene mutations on the prognosis of MDS patients. Univariate and multivariate Cox regression analyses and Lasso regression were used to screen for key prognostic factors. The shinyapps website was used to create dynamic nomograms with multiple variables. The time-dependent receiver operating characteristic (ROC) curves, calibration plots, and decision curve analysis (DCA) were used to evaluate the model’s discrimination, accuracy and clinical utility.

**Results:**

Six risk factors (age, bone morrow blast percentage, ETV6, TP53, EZH2, and ASXL1) were considered as predictor variables in the nomogram. The nomogram showed excellent discrimination, with respective the area under the ROC curve (AUC) values of 0.850, 0.839, 0.933 for the training cohort at 1 year, 3 years and 5 years; 0.715, 0.802 and 0.750 for the testing cohort at 1 year, 3 years and 5 years; and 0.668, 0.646 and 0.731 for the external validation cohort at 1 year, 3 years and 5 years. The calibration curves and decision curve showed that the nomogram had good consistency and clinical practical benefit. Finally, a stratified analysis showed that MDS patients with high risk had worse survival outcomes than patients with low risk.

**Conclusion:**

We developed a nomogram containing six risk factors, which provides reliable and objective predictions of prognosis for MDS patients.

## Introduction

Myelodysplastic syndrome (MDS) is a rare group of clonal myeloid malignancies characterized by ineffective hematopoiesis and high-risk progression to acute myeloid leukemia (AML) (1). The incidence of MDS increases with age, especially in patients over 70 years old (2, 3). Due to the heterogeneity of clinical manifestations, the survival time of patients ranges from approximately a few months to several years. Accurately estimating the prognosis of patients at primary diagnosis could allow patients to benefit from the management of disease and assist doctors in implementing precise treatment (4).

Currently, several prognostic scoring systems have been developed to stratify the risk of MDS patients, including the International Prognostic Scoring System (IPSS), Revised IPSS (IPSS-R), World Health Organization-based Prognostic Scoring System (WPSS) and Global MD Anderson Prognostic Scoring System (MDAPSS) (5, 6). These systems apply several variables, such as bone marrow blast percentage, cytogenetics and laboratory testing, which are widely used in clinical practice. With the maturation of high-throughput sequencing technology, somatic mutations have been accurately detected and found to play a crucial role in the clinical phenotype, prognosis, and response to therapy of MDS patients (7, 8). This advance has enabled our understanding of the genetics of MDS to leap from chromosomal abnormalities to somatic mutations at the genome-wide level (9). Therefore, the genetics of MDS urgently need to be reviewed to comprehensively analyze MDS disease-related factors and construct a novel prognostic model.

In this study, we aimed to develop and validate a novel prognostic model that will incorporate more molecular genetic features. Furthermore, the model will assist clinicians in easily and reliably evaluating the prognosis of individual MDS patients.

## Material and methods

### Data collection

Two GEO datasets [GSE58831 (10) and GSE129828 (11)] were downloaded from the Gene Expression Omnibus (GEO) database (https://www.ncbi.nlm.nih.gov/geo/) for model construction. Inclusion criteria for this study were definite pathologically diagnosed *de novo* myelodysplastic syndrome with complete clinical information and follow-up data. Study variables list as follow (1): patient admission status, such as age, gender, hemoglobin, absolute neutrophil count, platelet count, and percentage of bone marrow blast cell(BM%); (2) molecular biological examination includes karyotypes and gene mutations. The primary outcome measure was 1-year, 3-year and 5-year overall survival (OS) rate. Overall survival was defined as the time from initial diagnosis until death or censoring at the time of the last follow-up for patients last known to be alive. In addition, we also collected clinical information from 115 MDS patients who visited the First Hospital of Lanzhou University range from June 2015 to June 2022, which was used for external validation of the model. This study was approved by the Ethics Committee of the First Hospital of Lanzhou University.

### Variable selection

In the R software, the “createDataPartition” function in the “caret” package is used for random grouping. The entire GEO cohort is divided into a training cohort and a testing cohort at a 1:1 ratio. In the training cohort, univariate, lasso, and multivariate cox regression analyses were used to screen for key prognostic factors. Specifically, univariate, multivariate Cox regression analysis was performed using the “survival” package to identify prognostic-related variables. Lasso regression analysis using “glmnet” has solved the collinearity problem.

### Nomogram construction

The identified independent prognostic factors were constructed using the “regplot” package to construct a nomogram, where continuous variables were showed by line segments and categorical variables were showed by boxes. By matching each variable in the nomogram, we could calculate the corresponding score and total points. Correspondingly, the 1-year, 3-year, and 5-year survival rates of the patient were calculated. In addition, we have shared and deployed our nomogram on the shinyapps website (www.shinyapps.io) so that clinicians can easily and quickly apply the nomogram to assess patient outcomes.

### External validation of the nomogram

In the training cohort, the time-dependent receiver operating characteristic (ROC) curve was plotted using the “timeROC” package to evaluate the discriminativeness of the prediction model. Use the “rms” package to draw a calibration curve, and cycle sampling 1,000 times by the Bootstrap method to compare the closeness of the predicted survival rate to the actual survival rate, thereby judging the accuracy of the model. A decision curve analysis (DCA) was drawn using the “ggDCA” package to evaluate the net benefit of prognostic model in guiding clinical decision-making. In addition, the above metrics were also evaluated in the internal testing cohort, the entire cohort and the external validation cohort.

### Statistical analysis

All statistical analyses and graphs were performed using R software 4.1.2 (www.r-project.org) and Graph-pad Prism 8.3 software (San Diego, California, USA). For overall survival of patients in different groups were estimated by Kaplan-Meier methodology and Log-rank test.

## Results

### Patient characteristics and work flow

**Figure 1:**
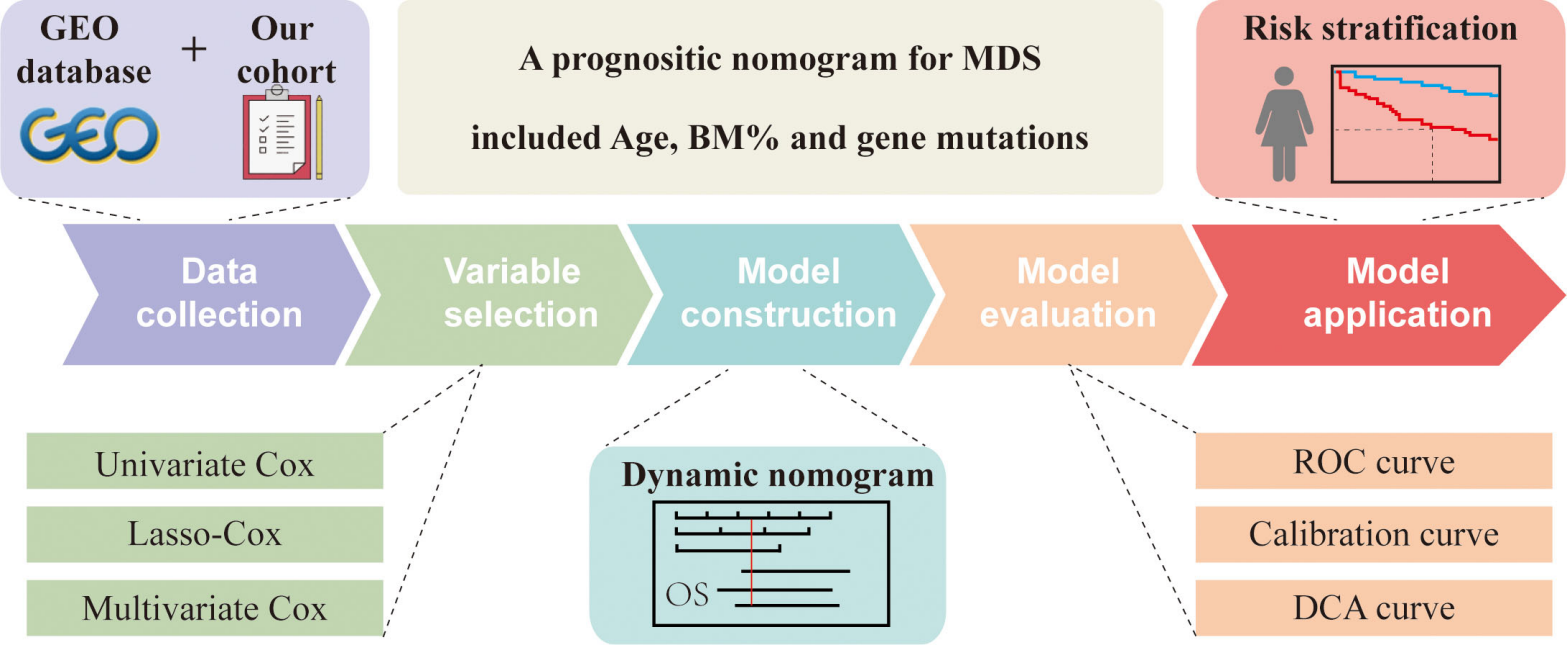
Flowchart of study.

In the present study, we screened 201 MDS patients with complete clinical information according to strict inclusion and exclusion criteria. The patients were randomly divided into two groups at a 1:1 ratio to explore the relationship between clinical variables and the prognosis of MDS patients. The training cohort included 101 MDS patients with a median survival time of 31 months, and the testing cohort included 100 MDS patients with a median survival time of 36.8 months. The clinical characteristics of the two groups of patients are shown in **Supplementary Table 1**. As the flowchart shown in **Figure 1**, we used univariate, lasso, and multivariate cox regression analyses to screen for key prognostic factors and construct a nomogram. Subsequently, ROC curves, calibration plots, and DCA analysis were used to evaluate the predictive performance of the model. Finally, we stratified patients using risk scores in all cohorts.

### Association between gene mutations and MDS patient prognosis

**Figure 2:**
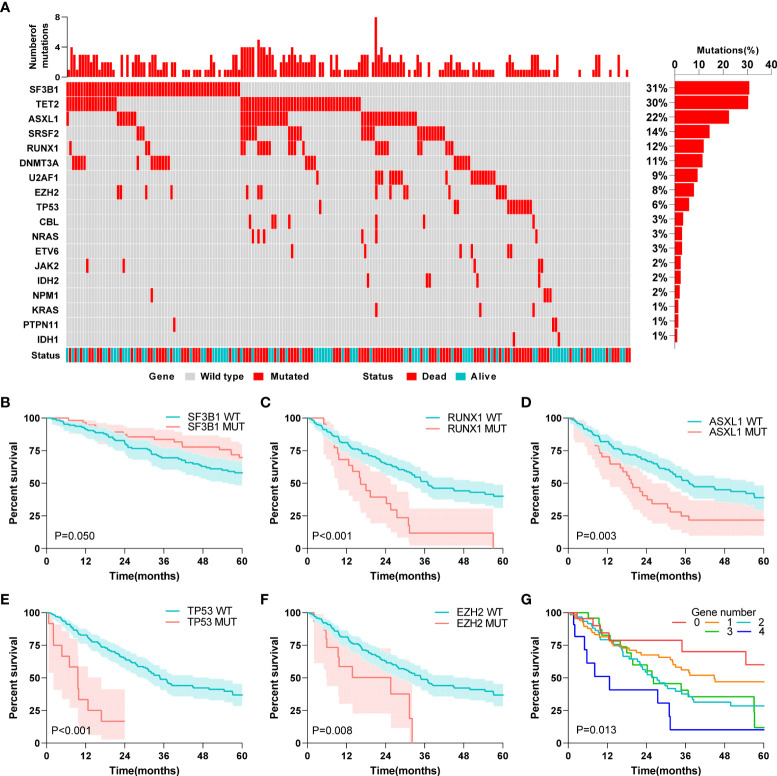
Association between gene mutations and MDS patients’ prognosis. **(A)** Landscape profile of 18 somatic gene mutations in 201 MDS patients. Mutations of each genes in each patient were shown in waterfall plot. Each column presented each patient. The left is the number of mutations in each patient, and the right is the mutation frequency of the gene in 201 patients. **(B)** Kaplan-Meier analysis of SF3B1 wild-type and mutant MDS patients. **(C)** Kaplan-Meier analysis of RUNX1 wild-type and mutant MDS patients. **(D)** Kaplan-Meier analysis of ASXL1 wild-type and mutant MDS patients. **(E)** Kaplan-Meier analysis of TP53 wild-type and mutant MDS patients. **(F)** Kaplan-Meier analysis of EZH2 wild-type and mutant MDS patients. **(G)** Kaplan-Meier analysis of MDS patients with different numbers of mutated genes. The log-rank test was used to compare survival rates between the two groups.

First, we describe the 18 MDS-related gene mutations in 201 MDS patients from the entire GEO cohort. Different patients showed a gross difference in mutation burden (**Figure 2A**). The number of gene mutations ranged from none to eight, and the vast majority (85%) of patients had fewer than three types of mutated genes. Specifically, SF3B1 was the most frequently mutated gene, with a mutation rate of 31%, followed by TET2 and ASXL1, with mutation rates of 30% and 22%, respectively. The mutation rates of KRAS, PTPN11, and IDH1 were the lowest (1%). The mutation status of the external validation cohort is also shown in **Supplementary Figure 1**. TET2 and ASXL1 still had higher mutation rates in the external validation cohort. A further survival analysis showed that five gene mutations were associated with the OS of patients with MDS, including the beneficial mutation SF3B1 (*P*=0.050), and four unfavorable mutations, RUNX1 (*P*<0.001), ASXL1 (*P*=0.003), TP53 (*P*<0.001) and EZH2 (*P*=0.008) (**Figures 2B–F**). Interestingly, we found that the number of mutations was also associated with prognosis (*P*=0.013) (**Figure 2G**), which fully suggested that the role of gene mutations in the prognosis of MDS patients should not be underestimated.

### Association between karyotype classification and MDS patient prognosis

**Figure 3:**
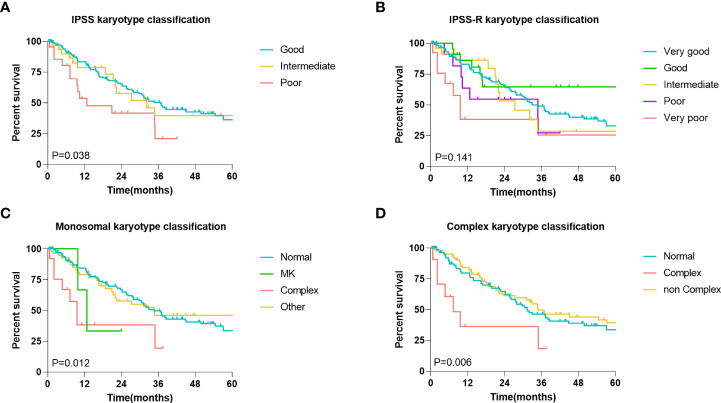
Association between karyotype classification and MDS patients’ prognosis. **(A)** Kaplan-Meier analysis of MDS patients with different IPSS karyotype classification. **(B)** Kaplan-Meier analysis of MDS patients with different IPSS-R karyotype classification. **(C)** Kaplan-Meier analysis of MDS patients with different monosomal karyotypes (MK) classification. **(D)** Kaplan-Meier analysis of MDS patients with different complex karyotypes (CK) classification. The log-rank test was used to compare survival rates between the two groups.

Subsequently, to clarify the influence of abnormal chromosomal karyotypes on the prognosis of MDS patients, we analyzed the relationship between different karyotype classifications and the prognosis of MDS patients. Four different representative karyotype classifications were selected to characterize karyotype-prognostic relationships. According to the IPSS karyotype classification, 72.6% of the patients had good karyotypes, 15.9% had moderate karyotypes, and 11.5% had poor karyotypes in the entire cohort. According to the IPSS-R karyotype classification, 62.2% of the patients had a very good karyotype, 12.4% had a good karyotype, 12.9% had a moderate karyotype, and 6.0% had a poor karyotype, and very poor karyotypes accounted for 6.5% of all patients. Considering the monosomal karyotype (MK), 61.7% of patients had a normal karyotype, 1.5% of patients had a MK, 6.5% of patients had a complex karyotype, and 31.3% of other patients had a normal karyotype. Considering only complex karyotypes (CK), 72.6% of patients had normal karyotypes, 15.9% of patients had complex karyotypes, and 11.5% of patients had noncomplex karyotypes. A further Kaplan-Meier survival analysis showed that in the entire cohort, all classifications other than the IPSS-R classification were related to prognosis (*P*<0.05, **Figures 3A–D**).

### Screening for prognosis-related variables

**Figure 4:**
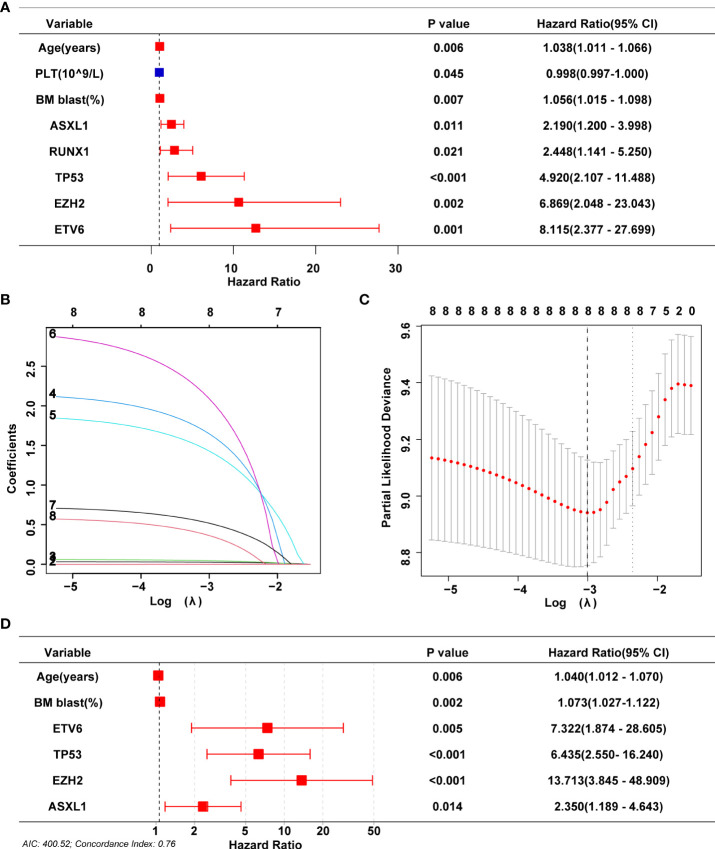
Screening for prognosis-related variables. **(A)** Univariate Cox analyses on variables for the prediction of overall survival of MDS patients in training cohort. Graph only shows significant results (P<0.05). **(B, C)** LASSO-Cox regression by 10-fold cross-validation was performed to select key variables. **(D)** Multivariate Cox proportional hazards were used for identifying independent prognostic variables.

To build the model, the entire cohort was randomly divided into a training cohort and a validation cohort. A univariate Cox regression model was performed to identify all prognostic risk factors(gender, age, hemoglobin, absolute neutrophil count, platelet count, percentage of bone marrow blast cell, 18 MDS-related gene mutations, IPSS karyotype classification, IPSS-R karyotype classification, MK karyotype classification, and CK karyotype classification) in the training cohort. Meanwhile, we again evaluated the impact of different karyotypes on patient prognosis in the training cohort (**Supplementary Figures 2A–D**) and validation cohort (**Supplementary Figures 2E–H**) to comprehensively identify key feature. Eight characteristics, including age, platelets, BM blast percentage, and mutations in ASXL1, EZH2, RUNX1, ETV6 and TP53, were associated with MDS patient OS (**Figure 4A**, **Supplementary Table 2**). A Lasso-Cox regression analysis further retained these eight features for subsequent analysis (**Figures 4B, C**). Finally, age, BM blast percentage, and ASXL1, EZH2, ETV6, and TP53 mutations were identified as key independent prognostic factors by a multivariate Cox regression analysis (**Figure 4D**).

### Nomogram and dynamic nomogram MDS for OS

**Figure 5:**
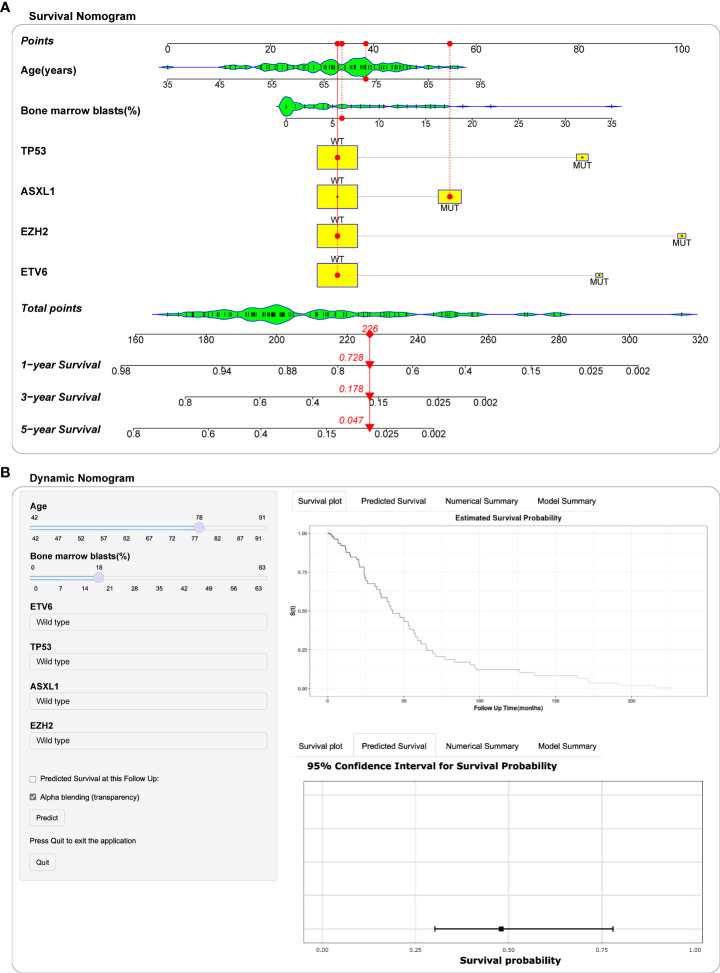
Construction of nomogram and dynamic nomogram. **(A)** Nomogram for predicted 1-year, 3-year and 5-year survival probability for MDS patients. A 73-year-old female patient had a bone marrow blast percentage of 6% with ASXL1 mutation and ETV6 mutation. According to the nomogram, the patient has a risk score of 226, which determines her survival probability of 1 year (72.8%), 3 years (17.8%) and 5 years (4.7%), respectively. **(B)** A dynamic Nomogram to predict the survival of MDS patients in Shinyapps website.

Based on the above screening analysis, we established a prognostic nomogram with six variables, including age, BM blast percentage, ASXL1, EZH2, ETV6 and TP53 mutations (**Figure 5A**). In the nomogram, each variable is matched to a specific score by weight, and the sum of all scores corresponds to the predicted probability of 1-year, 3-year and 5-year overall survival. For example, a 73-year-old female patient with a BM blast percentage of 6% and genetic assay showed that she harbored an ASXL1 mutation. According to the nomogram, the patient has a risk score of 226, which determines her 1-year (72.8%), 3- year (17.8%) and 5-year (4.7%) probability of survival (**Figure 5A**). Moreover, we deployed the nomogram on the shinyapps website (https://seeapple.shinyapps.io/MDS_Nomogram/), which allows clinicians to conveniently and directly utilize our model in real time (**Figure 5B**).

### Model validation and performance

**Figure 6:**
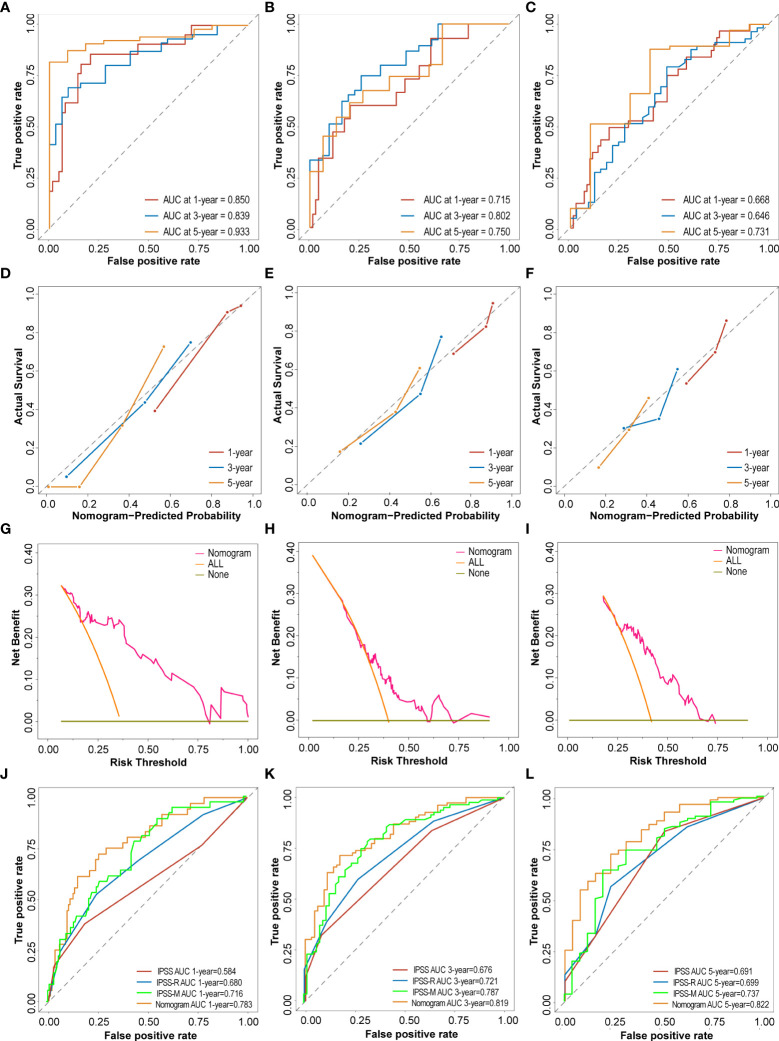
Performance and validation of the Nomogram. **(A–C)** The time-dependent receiver operating characteristic (ROC) curve and the area under the ROC curve (AUC) of nomogram at 1 year, 3 years and 5 years in the training cohort, testing cohort and external validation cohort. **(D–F)** The calibration plots of nomogram for predicting the 1 year, 3 years and 5 years in the training cohort, testing cohort and external validation cohort. **(G–I)** The Decision curve analysis (DCA) of nomogram for predicting the 5 years OS in the training cohort, testing cohort and external validation cohort. **(J–L)** ROC curves and the AUC of IPSS score, IPSS-R score, IPSS-M score and nomogram at 1 year, 3 years and 5 years in the entire GEO cohort.

To evaluate and verify the performance of the nomogram, ROC curves, calibration curves and DCA analysis were performed for each cohort. In the training cohort, the nomogram concordance index (C-index) for predicting OS was 0.763. The area under the ROC curve (AUC) values for the 1-year, 3-year, and 5-year OS were 0.850 (95%CI, 75.23 to 94.80), 0.839 (95%CI, 75.36 to 92.52) and 0.933 (95%CI, 87.39 to 99.21), respectively (**Figure 6A**). Subsequently, the predictive accuracy was measured in the testing cohort. C-index was 0.678, and AUC values for the 1-year, 3-year, and 5-year OS were 0.715 (95%CI, 56.96 to 86.12), 0.802 (95%CI, 70.11 to 90.26) and 0.750 (95%CI, 61.51 to 88.28), respectively (**Figure 6B**). Meanwhile, the favorable calibration of the nomogram for the predicted probabilities of 1-year, 3-year, and 5-year OS was observed both in the training and internal testing cohorts, with good correlations between the predicted and observed survival proportions at different time points (**Figures 6D, E** **)**. In addition, the external validation cohort included 115 patients from a single-center cohort in China northwestern for the final evaluation. The nomogram C-index for predicting OS was 0.639. The AUC values for the 1-year, 3-year, and 5-year OS were 0.668 (95%CI, 55.70 to 77.99), 0.646 (95%CI, 52.53 to 76.66) and 0.731 (95%CI, 55.06 to 91.08), respectively (**Figure 6C**). Moreover, the external calibration plots for the predicted probabilities of 1 year, 3 years, and 5 years OS also showed adequate consistency (**Figure 6F**). The DCA analysis of the nomogram was higher than that of the None and All lines in training cohort, internal testing cohort and external validation cohort, indicating that the nomogram would be more beneficial (**Figures 6G–I**). Compared to the IPSS and IPSS-R, the nomogram achieved an improved AUC at 1 year, 3 years and 5 years in the entire GEO cohort (1 year: 0.783 (95%CI, 70.01 to 86.64) vs. 0.584 (95%CI, 47.14 to 69.67) vs. 0.680 (95%CI, 58.14 to 77.87); 3 years: 0.819 (95%CI, 75.29 to 88.57) vs. 0.676 (95%CI, 60.01 to 75.22) vs. 0.721 (95%CI, 64.33 to 79.85); 5 years: 0.822 (95%CI, 73.45 to 90.98) vs. 0.691 (95%CI, 58.38 to 79.85) vs. 0.699 (95%CI, 59.12 to 80.72); **Figures 6J–L**). A recent study reported a clinical-molecular prognostic model (IPSS-Molecular [IPSS-M]) for risk stratification in patients with MDS (12). By comparing the IPSS-M score with nomogram of present study, our model was slightly non-inferior to IPSS-M in predicting patients 1 year, 3 years and 5 years OS (**Figures 6J–L**).

### Risk stratification

**Figure 7:**
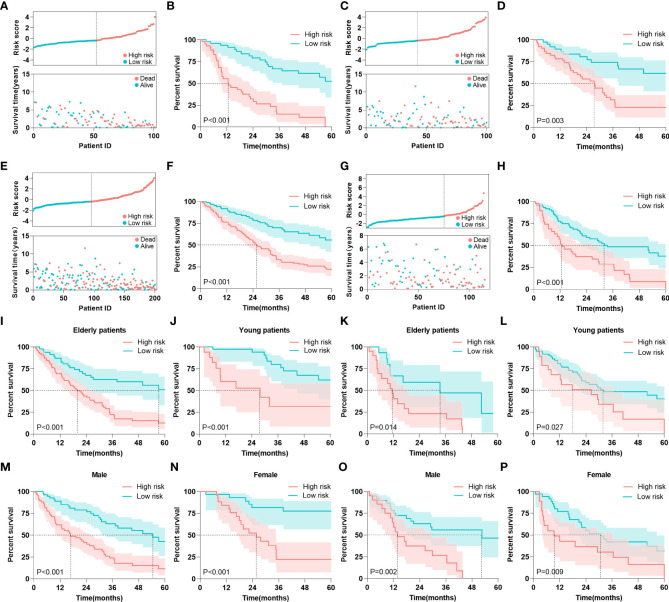
Risk stratification. **(A)** Risk scores and survival times of patients were in the training cohort. **(B)** Kaplan-Meier analysis of MDS patients with different risk level in the training cohort. **(C)** Risk scores and survival times of patients were in the internal testing cohort. **(D)** Kaplan-Meier analysis of MDS patients with different risk level in the internal testing cohort. **(E)** Risk scores and survival times of patients were in the entire GEO cohort. **(F)** Kaplan-Meier analysis of MDS patients with different risk level in the entire GEO cohort. **(G)** Risk scores and survival times of patients were in the external validation cohort. **(H)** Kaplan-Meier analysis of MDS patients with different risk level in the external validation cohort. **(I, J)** Kaplan-Meier analysis of elderly and young patients with different risk level in the entire GEO cohort. **(K, L)** Kaplan-Meier analysis of elderly and young patients with different risk level in the external validation cohort. **(M, N)** Kaplan-Meier analysis of male and female patients with different risk level in the entire GEO cohort. **(O, P)** Kaplan-Meier analysis of male and female patients with different risk level in the external validation cohort. The log-rank test was used to compare survival rates between the two groups.

Based on the total points calculated by the nomogram, we divided all cohorts into high-risk and low-risk groups based on the median risk score in the training cohort (cut off by -0.37). In the training cohort, the risk score scatterplot showed that most of the patients who died were distributed in the high-risk group, while the Kaplan-Meier analysis showed that high-risk patients had lower 5 years survival rates (54% vs 0%) (**Figures 7A, B** **)**. In addition, risk scores were effective in stratifying patients in the internal testing cohort (**Figures 7C, D** **)**, the entire cohort (**Figures 7E, F** **)** and the external cohort (**Figures 7G, H** **)**. In the entire GEO cohort, elderly patients (over 65 years old) with MDS were divided into high-risk and low-risk groups, and high-risk patients had lower 5 years survival rates (51% vs 13%) (**Figure 7I**). In addition, the model performed equally well in the risk classification of young MDS patients (**Figure 7J**). Moreover, the model achieved good risk stratification for elderly and young patients in the external validation cohort (**Figures 7K, L**). In terms of gender, the model was applicable in both the GEO cohort and the external validation cohort in both male and female patients (**Figures 7M–P**).

## Discussion

In the present study, we performed an integrative analysis of clinical, disease, and biological variables to identify MDS prognosis-associated characteristics, and constructed a dynamic nomogram. In total, we collected publicly accessible cases from 201 patients with MDS. The correlations between chromosomal abnormalities and gene mutations and the prognosis of patients with MDS were analyzed and compared. Subsequently, all patients were divided into the training and testing cohorts. Univariate and multivariate Cox regression analyses and LASSO regression were performed to select key prognostic variables, and a web-based dynamic nomogram that included patient age, BM blast cell percentage, ETV6, TP53, EZH2, and ASXL1 mutations was constructed simultaneously. Receiver operating characteristic (ROC) curves, calibration plots, and DCA analyses were used to evaluate the predictive performance and applicability of the nomogram. In addition, this model externally validated using a single-center cohort of 115 patients with MDS.

Age is the primary risk factor for the development of MDS, with the median age at diagnosis exceeding 70 years. The interaction of diseases, comorbidities, and frailty results in poor prognoses in elderly patients, which can be seen in most diseases, especially in patients with cancer (13). The percentage of blast cells has long been considered a major criterion for the diagnosis of MDS, and serves as the basis for prognostic classification. A higher blast cell percentage is thought to reflect a greater disease burden and more advanced disease, and is associated with worse outcomes (14, 15). Consistent with our findings, when we constructed a prognostic model, age and BM blast percentage remained the essential features that we prioritized and screened.

With the advancement of sequencing technology, next-generation sequencing (NGS) has become a popular method for the diagnosis of MDS. Specific genes involved in epigenetic regulation (TET2, ASXL1, EZH2, DNMT3A, and IDH1/2), RNA splicing (SF3B1, SRSF2, U2AF1, and ZRSR2), DNA damage response (TP53), transcriptional regulation (RUNX1, BCOR, and ETV6), and signal transduction (CBL, NRAS, and JAK2) have been identified in MDS (16, 17). More than 90% of patients with MDS harbor somatic myeloid-related mutations (18, 19). In this study, four important genes, TP53, ASXL1, EZH2, and ETV6, were selected to construct a nomogram. Tumor protein 53 (TP53) is a tumor suppressor gene that has received considerable attention in all types of tumors. TP53 mutations are strongly associated with rapid transformation of high-risk MDS into AML, resistance to conventional therapy, and poor outcomes (20). Based on these, TP53-mutant MDS was once called a “black hole of hematology” (21). A recent study of 3324 patients with MDS reported that patients with TP53 mutations have both monoallelic (1/3) and biallelic mutations (2/3). Interestingly, monoallelic mutations did not differ from those of TP53 wild-type patients in terms of outcome and response to treatment (22). In contrast, previously reported complex karyotypes, rare co-occurring mutations, high-risk manifestations, and poor outcomes were strongly associated with biallelic mutations. This finding is largely attributable to the fact that monoallelic mutations need to cooperate with other driver mutations or secondary TP53 dysfunction to maintain malignancy, which alone is not sufficient to cause MDS (23). Additional sex comb-like 1 (ASXL1) plays a crucial role in epigenetic regulation, including the modification of histone methylation and regulation of transcription of genes involved in differentiation and proliferation. ASXL1 mutations are usually associated with distinct epigenomic alterations that increase the sensitivity of patients with MDS to venetoclax and azacytidine (24). ASXL1 mutations are frequent epigenetic regulatory aberrations in MDS that predict adverse prognostic outcomes. Thus, screening patients for ASXL1 mutations may be useful for clinical risk stratification and treatment decisions in the future (25). Mutations in the enhancer of zeste homolog 2 (EZH2) gene are frequently affected by abnormalities in chromosome 7, which is frequently detected in myeloid malignancies (26). EZH2 is the core component of the polycomb repressive complex 2 (PRC2), which is responsible for gene silencing by posttranslational histone modifications (27). EZH2 mutations are associated with oncogenesis and progression, and predict poor prognosis in patients with myeloid neoplasms (28). As a tumor suppressor gene, ETS variant transcription factor 6 (ETV6) plays an important role in hematopoietic stem cell maintenance and lineage differentiation, participates in oncogenic fusion, and regulates thrombopoiesis (29). Rearrangement is a common form of ETV6 mutation, and ETV6 can form fusion genes with ARNT, MN1, ACS2, EVI1/MDS1, and JAK2. A higher ETV6 rearrangement rate is closely related to the disease stage and prognosis of MDS (30).

Although current clinical risk stratification tools perform well in predicting the prognosis of patients with MDS, neither IPSS nor IPSS-R consider somatic mutations (31). NGS in patients with MDS is now a routine laboratory test for physicians. Similar to the emergence of ChromoSeq technology, whole-genome sequencing will gradually replace traditional cytogenetic testing, especially for the detection of mysterious and rare chromosomal abnormalitie (32). Therefore, consideration of genetic mutations in the prognostic analysis of MDS is overwhelming. By comparing the area under the ROC curve, we found that the area under the nomogram was superior to those of both IPSS and IPSS-R in predicting 1, 3, and 5 years patient survival status.

In recent years, several similar studies have also focused on the risk stratification of patients with MDS, such as the IPSS-M and Aziz Nazha’s model (12, 33). The IPSS-M included hematologic parameters, cytogenetic abnormalities, and somatic mutations of 31 genes, emphasizing the importance of gene mutations in the prognosis of patients with MDS. However, the nomogram was noninferior to the IPSS-M in predicting the survival of patients with MDS. Nazha et al. proposed an integrated IPSS-R scoring system and mutational data, where IPSS-R has a smaller proportion than gene mutations. Based on this study, we replaced IPSS-R with the percentage of blast cells, which improved the performance of the model. Nevertheless, large-scale, multicenter cohorts are still needed to verify the applicability of the results. In addition, some gene signatures to predict the prognosis of patients with MDS have also been reported, such as autophagy gene signatures by Hu et al. and metabolic gene signatures by Liang et al. (34, 35). The nomogram in this study performed considerably better than the previous signatures in discriminating power using only readily available information on four genetic mutations.

This study had several advantages. First, our study strongly emphasizes the role of genetic mutations in the prognosis of MDS compared to previous prognostic models. Second, we developed and validated the model using MDS cohorts from the United Kingdom and the United States, and supplied an MDS cohort from a single center in Northwest China for external validation, which greatly improved the applicability of the predictive model. Most importantly, we uploaded the nomogram to a website that can be easily accessed by physicians and patients through internet terminals.

This study had some limitations. First, owing to the limited number of study patients, some low-frequency, functionally important gene mutations may have been overlooked. Moreover, the public data did not specify the type of gene mutation, and the role of gene mutations in prognosis could not be deeply explored. Second, because the three centers lacked a unified pathological classification for MDS, pathological classification was not considered in this study. Third, our cohort was retrospective; thus, it was inevitably affected by selection and recall bias. In addition, we will also consider treatment-related factors and leukemia transformation, and analyze the relationship between risk groups and treatment strategies in future studies.

In conclusion, we developed a nomogram containing six risk factors that provides reliable and objective predictions of the prognoses of patients with MDS. Importantly, a dynamic nomogram is a functional and practical tool that can help clinicians assess patient prognosis and determine appropriate treatment strategies.

## Data availability statement

The datasets presented in this study can be found in online repositories. The names of the repository/repositories and accession number(s) can be found in the article/**Supplementary Material.**

## Ethics statement

The studies involving human participants were reviewed and approved by the Ethics Committee of the First Hospital of Lanzhou University. The patients/participants provided their written informed consent to participate in this study.

## Author contributions

All authors of this study have directly participated in the planning, execution, or analysis of the study. HL and YF were involved in the acquisition, analysis, and interpretation of the data and drafting of the manuscript. JJ, XL and LT were involved in data collection. YG was involved in the analysis and interpretation of data. LZ and BL were involved in the conception and design of the study, and critically revising the manuscript and overall study supervision. All authors contributed to the article and approved the submitted version.

## Acknowledgments

We thank the patients and investigators who submitted in GEO database for providing high quality data analyzed in this study. Thanks to the patients who provided clinical data, and the medical testing institutions that provided support.

## Conflict of interest

The authors declare that the research was conducted in the absence of any commercial or financial relationships that could be construed as a potential conflict of interest.

## Publisher’s note

All claims expressed in this article are solely those of the authors and do not necessarily represent those of their affiliated organizations, or those of the publisher, the editors and the reviewers. Any product that may be evaluated in this article, or claim that may be made by its manufacturer, is not guaranteed or endorsed by the publisher.

## Abbreviation

AML, acute myeloid leukemia; ASXL1, Additional sex combs-like 1; AUC, the area under the ROC curve; BM, bone marrow; CK, complex karyotype; DCA, Decision curve analysis; ETV6, ETS variant transcription factor 6; EZH2, Enhancer of Zeste Homolog 2; GEO, the Gene Expression Omnibus; IPSS, International Prognostic Scoring System; IPSS-R, Revised IPSS; MDS, Myelodysplastic Syndrome; NGS, next generation sequencing; ROC, the time-dependent receiver operating characteristic; MDAPSS, the Global MD Anderson Prognostic Scoring System; MK, Monosomal karyotype; NGS, next generation sequencing; TP53, Tumor protein 53; WPSS, World Health Organization based Prognostic Scoring System.
