# Supplementary material for: Development and validation of a novel prognosis prediction model for patients with myelodysplastic syndrome
Source: Front Oncol. 2022 Oct 12;12:1014504. doi: 10.3389/fonc.2022.1014504 (PMC9597308; doi:10.3389/fonc.2022.1014504)
Supplement: Supplementary file 1 [file DataSheet_1.docx]

Supplementary Material

# Supplementary Figures and Tables

## Supplementary Figures

**
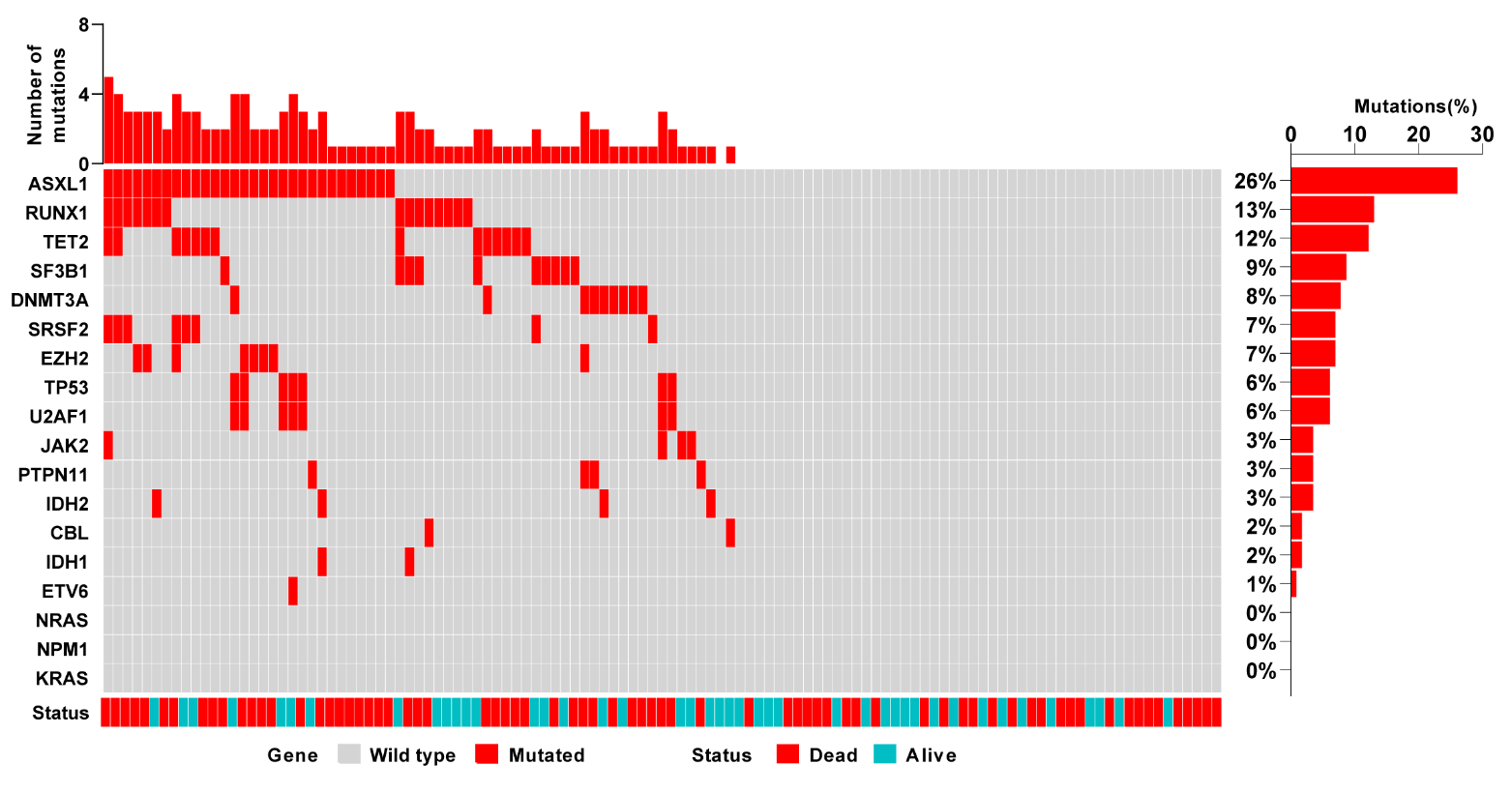
**

**Supplementary Figure 1.** Landscape profile of basic clinical information and 18 somatic gene mutations in 115 MDS patients of a single-center cohort. Mutations of each genes in each patient were shown in waterfall plot. Each column presented each patient.


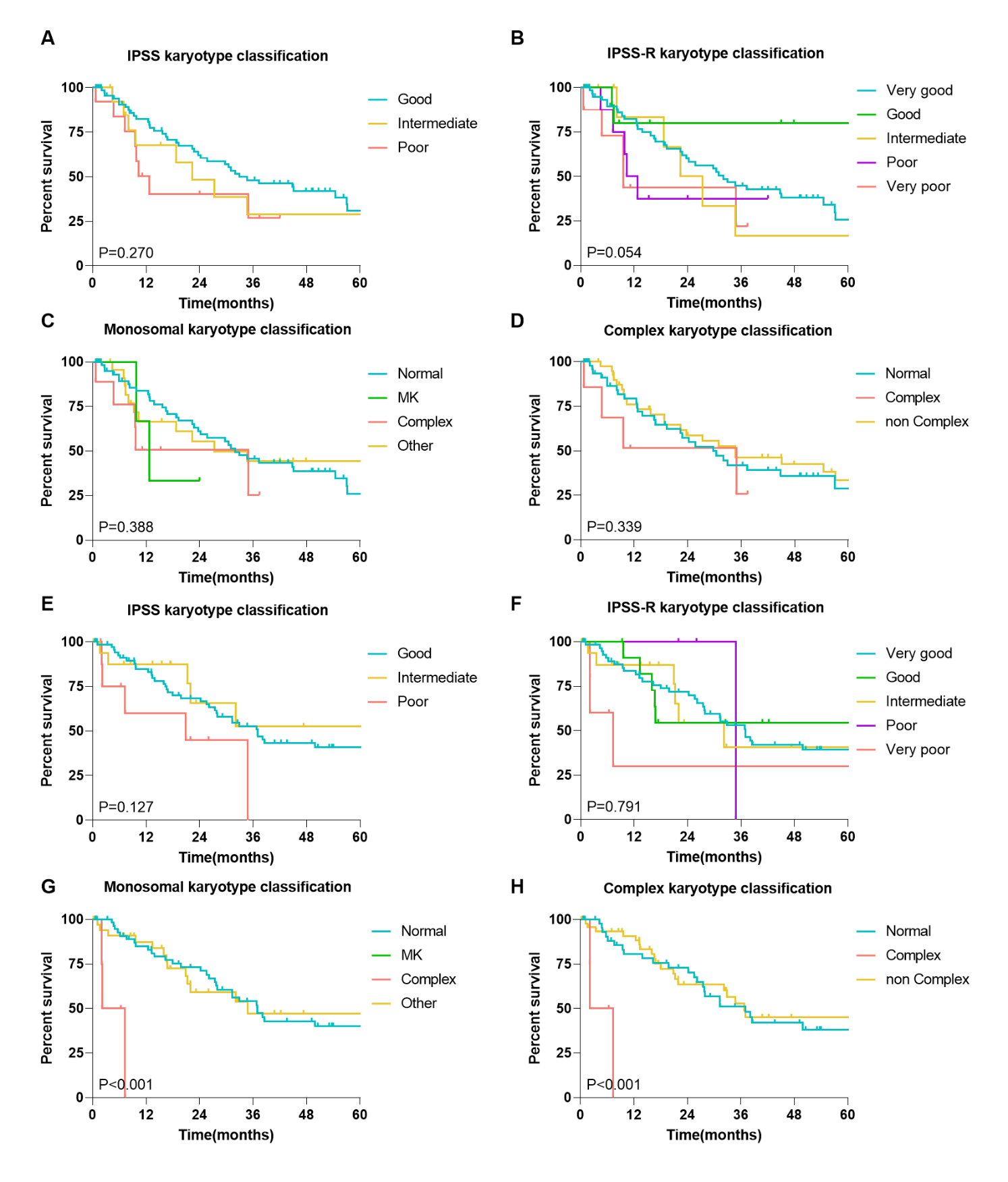
**Supplementary Figure 2 Association between karyotype classification and MDS patients’ prognosis**

(A-D) Kaplan-Meier analysis of MDS patients with different IPSS, IPSS-R, monosomal, complex karyotype classification in the training cohort. (E-H) Kaplan-Meier analysis of MDS patients with different IPSS, IPSS-R, monosomal, complex karyotype classification in the testing cohort.

## Supplementary Tables

**Supplementary Table 1.** Clinical information of MDS patients from GEO and a single-center Northwest China cohort.

| Characteristic | | GEO cohort | | | Northwest China cohort  (n=115) |
| --- | --- | --- | --- | --- | --- |
|  |  | Training cohort  (n=101) |  | Testing cohort  (n=100) |  |
| Age (years) | | 67.55±10.22 |  | 68.90±11.92 | 57.31±13.89 |
| Gender | Male | 74(73.27%) |  | 68(68.00%) | 61(53.04%) |
|  | Female | 27(26.73%) |  | 32(32.00%) | 54(46.96%) |
| Hb (g/L) | | 9.85±1.82 |  | 9.66±1.66 | 7.30±2.65 |
| ANC (10^9/L) | | 2.90±3.28 |  | 2.94±3.76 | 2.43±4.95 |
| PLT (10^9/L) | | 198.23±197.24 |  | 166.36±157.50 | 92.78±115.92 |
| BM% | | 5.52±6.47 |  | 6.02±8.24 | 4.21±6.32 |
| IPSS Good | | 73(72.28%) |  | 73(73.00%) | 88(76.52%) |
| Intermediate | | 15(14.85%) |  | 17(17.00%) | 17(14.78%) |
| Poor | | 13(12.87%) |  | 10(10.00%) | 10(8.70%) |
| IPSS-R Very good | | 65(64.36%) |  | 60(60.00%) | 2(1.74%) |
| Good | | 11(10.89%) |  | 14(14.00%) | 87(75.65%) |
| Intermediate | | 9(8.91%) |  | 17(17.00%) | 16(13.91%) |
| Poor | | 8(7.92%) |  | 4(4.00%) | 4(3.48%) |
| Very poor | | 8(7.92%) |  | 5(5.00%) | 6(5.22%) |
| MK Normal | | 64(63.37%) |  | 59(59.00%) | 82(71.30%) |
| MK | | 3(2.97%) |  | 0(0%) | 3(2.61%) |
| CK | | 9(8.91%) |  | 4(4.00%) | 7(6.09%) |
| Other | | 25(24.75%) |  | 37(37.00%) | 23(20.00%) |
| CK Normal | | 52(51.49%) |  | 47(47.00%) | 82(71.30%) |
| CK | | 7(6.93%) |  | 4(4.00%) | 7(6.09%) |
| Non CK | | 42(41.58%) |  | 49(49.00%) | 26(22.61%) |
| Status | Alive | 43(42.57%) |  | 48(48.00%) | 43(37.39%) |
|  | Dead | 58(57.43%) |  | 52(52.00%) | 72(62.61%) |

**Supplementary Table 2.** Prognostic factors in the univariate Cox analysis for MDS patients in the training cohort.

| Variable | HR | 95% CI | P-value |
| --- | --- | --- | --- |
| Gender(Female vs Male) | 0.542 | 0.288 - 1.018 | 0.057 |
| Age(years) | 1.038 | 1.011 - 1.066 | 0.006 |
| HB(g/dL) | 0.957 | 0.821 - 1.117 | 0.580 |
| ANC(10^12/L) | 1.055 | 0.965 - 1.154 | 0.241 |
| PLT(10^9/L) | 0.998 | 0.997 - 1 | 0.045 |
| BM blast(%) | 1.056 | 1.015 - 1.098 | 0.007 |
| NRAS(MUT vs WT) | 2.745 | 0.660 - 11.418 | 0.165 |
| SRSF2(MUT vs WT) | 1.642 | 0.822 - 3.280 | 0.160 |
| KRAS(MUT vs WT) | 0 | 0 – Inf | 0.997 |
| ETV6(MUT vs WT) | 8.115 | 2.377 - 27.699 | 0.001 |
| JAK2(MUT vs WT) | 0.365 | 0.050 - 2.646 | 0.318 |
| CBL(MUT vs WT) | 0.685 | 0.166 - 2.820 | 0.600 |
| DNMT3A(MUT vs WT) | 0.958 | 0.409 - 2.243 | 0.921 |
| SF3B1(MUT vs WT) | 0.665 | 0.373 - 1.185 | 0.166 |
| TP53(MUT vs WT) | 4.920 | 2.107 - 11.488 | ＜0.001 |
| EZH2(MUT vs WT) | 6.869 | 2.048 - 23.043 | 0.002 |
| ASXL1(MUT vs WT) | 2.190 | 1.200 - 3.998 | 0.011 |
| IDH1(MUT vs WT) | 0.785 | 0.108 - 5.694 | 0.811 |
| PTPN11(MUT vs WT) | 0 | 0 – Inf | 0.996 |
| TET2(MUT vs WT) | 0.900 | 0.519 - 1.560 | 0.708 |
| RUNX1(MUT vs WT) | 2.448 | 1.141 - 5.250 | 0.021 |
| U2AF1(MUT vs WT) | 1.701 | 0.523 - 5.532 | 0.377 |
| IDH2(MUT vs WT) | 1.388 | 0.191 - 10.117 | 0.746 |
| NPM1(MUT vs WT) | 0 | 0 - Inf | 0.996 |
| Mutated gene number | 1.293 | 0.996 - 1.678 | 0.054 |
| MK(MK vs Normal) | 1.843 | 0.436 - 7.784 | 0.406 |
| MK(CK vs Normal) | 1.768 | 0.688 - 4.541 | 0.237 |
| MK(Other vs Normal) | 0.778 | 0.404 - 1.499 | 0.453 |
| IPSS-R(Good vs Very good) | 0.290 | 0.089 - 0.953 | 0.041 |
| IPSS-R(Intermediate vs Very good) | 1.153 | 0.452 - 2.942 | 0.766 |
| IPSS-R(Poor vs Very good) | 1.863 | 0.720 - 4.816 | 0.199 |
| IPSS-R(Very poor vs Very good) | 2.043 | 0.795 - 5.248 | 0.138 |
| IPSS(Intermediate vs Good) | 1.059 | 0.493 - 2.273 | 0.884 |
| IPSS(Poor vs Good) | 1.870 | 0.865 - 4.046 | 0.112 |
| CK(CK vs Normal) | 1.612 | 0.564 - 4.606 | 0.372 |
| CK(Non-CK vs Normal) | 0.773 | 0.449 - 1.330 | 0.353 |
